# Advantages and Prospects of Tag/Catcher Mediated Antigen Display on Capsid-Like Particle-Based Vaccines

**DOI:** 10.3390/v12020185

**Published:** 2020-02-06

**Authors:** Kara-Lee Aves, Louise Goksøyr, Adam F. Sander

**Affiliations:** 1 Faculty of Health Science, Institute for Immunology and Microbiology, University of Copenhagen, 2200 Copenhagen, Denmark; kara-lee@sund.ku.dk (K.-L.A.); louiseg@sund.ku.dk (L.G.); 2 AdaptVac Aps, Agern Alle 1, 2970 Hørsholm, Denmark

**Keywords:** capsid, antigen display, virus-like particle, vaccine, platform

## Abstract

Capsid-like particles (CLPs) are multimeric, repetitive assemblies of recombinant viral capsid proteins, which are highly immunogenic due to their structural similarity to wild-type viruses. CLPs can be used as molecular scaffolds to enable the presentation of soluble vaccine antigens in a similar structural format, which can significantly increase the immunogenicity of the antigen. CLP-based antigen display can be obtained by various genetic and modular conjugation methods. However, these vary in their versatility as well as efficiency in achieving an immunogenic antigen display. Here, we make a comparative review of the major CLP-based antigen display technologies. The Tag/Catcher-AP205 platform is highlighted as a particularly versatile and efficient technology that offers new qualitative and practical advantages in designing modular CLP vaccines. Finally, we discuss how split-protein Tag/Catcher conjugation systems can help to further propagate and enhance modular CLP vaccine designs.

## 1. Rationale for the Development of Capsid-Like Particle-Based Vaccines

The development of vaccines remains the most effective method for preventing and controlling the spread of infectious diseases [1,2]. Traditional live-attenuated vaccines, such as those targeting measles [3], rubella [4] and smallpox [5] are highly immunogenic and can induce potent and long-lived antibody responses even after a single immunization [6]. This was originally ascribed to the ability of the attenuated virus to replicate in the host after vaccination. The high immunogenicity of live-attenuated viruses comes with a cost of increased safety risks and challenging manufacturing processes [7,8]. In contrast, modern recombinant subunit vaccines (i.e., based on a soluble protein antigen) show high safety, but in general fail to induce similar long-lasting antibody responses in humans [6,9,10,11,12]. Within recent years, several virus-like particle (VLP) based vaccines have been tested in clinical trials, of which vaccines targeting Hepatitis B (HBV) (Recombivax HB^®^ and Engerix-B^®^), Human papillomavirus (HPV) (Cervarix^®^, Gardasil^®^, and Gardasil 9^®^) and Hepatitis E (HEV) (Hecolin^®^) have been licensed [13]. Notably, the HPV vaccine stands as a unique example of a recombinant subunit vaccine with comparable immunogenicity to live-attenuated vaccines, even after a single dose [14,15,16]. This vaccine is formed by the self-assembly of the HPV major capsid protein into capsid-like particles (CLP), the structure of which is thought to be key to its high potency [17]. CLPs constitute a subclass of VLPs and are rigid, non-lipid, protein-based particles. A large number of studies have jointly established a strong causal link between the high immunogenicity of CLPs and their structural similarities to native viruses. Of these properties, their size (20–200 nm in diameter) and repetitive surface geometry are considered the most important [18,19,20,21,22,23,24,25,26]. Moreover, it has long been recognized that the immunogenicity of a vaccine antigen can be significantly increased if it is delivered to the immune system in a similar multivalent, repetitive and particulate format [27,28]. Consequently, several strategies have been pursued, exploiting CLPs as scaffolds for the presentation of heterologous antigens, including self-antigens.

Here, we will review key attributes of various CLP-based vaccine technologies in terms of their ability to facilitate a highly immunogenic epitope display. Additionally, practical aspects of the conjugation systems, such as their versatility, manufacturability and scalability will be discussed. In this context, we highlight the Tag/Catcher-AP205 platform as a particularly versatile and effective technology and provide a rationale for further development of this technology for vaccine design.

## 2. Methods for Antigen Display on CLPs

### 2.1. Genetic Fusion

The first widely used approach for CLP-based antigen display was by genetic fusion of a heterologous antigen sequence to the capsid subunit protein [27,28,29]. When successful, each subunit of the CLP will display the inserted antigenic sequence in a pattern matching that of the underlying CLP. Although this approach can deliver foreign epitopes in a viral-like configuration, it is generally limited to small peptide antigens that do not inhibit particle assembly [30,31,32]. However, in some cases, even small peptide antigens prevent CLP assembly. Thus, the success of genetic fusion is difficult to predict, and structural characteristics of the CLP as well as biochemical properties of the peptide must be taken into account [33,34]. Studies on HBcAg CLPs show that highly charged and hydrophobic peptides tend to prevent CLP assembly [30,35]. Due to the small size of the incorporated antigen, CLP vaccines made by genetic fusion generally induce narrow epitope-specific antibody responses [36]. The challenges associated with genetic fusion have to some extent been relieved by creating mosaic CLPs, consisting of both native and genetically modified CLP subunits. However, this is at the cost of reduced antigen density [37,38].

### 2.2. Modular Antigen Display

CLP-display of large and complex protein antigens is not easily obtained by genetic fusion. Thus, an alternative strategy has been to attach protein antigens to the surface of preassembled CLPs. Importantly, when using such modular approaches, the resultant epitope display is directly dependent on the applied conjugation method, as well as the specific surface geometry of the employed CLP backbone. In addition, these technologies allow for separate recombinant production of antigens in various expression systems, ensuring high quality and correct protein processing before CLP conjugation [39,40,41,42]. The different conjugation systems used for modular CLP vaccine development is described in detail elsewhere [43]. Here, the main techniques are listed in Table 1 to provide a comparative overview of their relative practicality and ability to facilitate a high-quality epitope display. This evaluation is done in recognition that HPV CLPs represent a highly immunogenic viral epitope display, and on that basis, modular platforms should likewise facilitate ordered, high-density and unidirectional presentation of antigens in their native conformation. This cannot, to a similar degree, be achieved by all available antigen conjugation methods.

#### 2.2.1. Chemical Conjugation and Click Chemistry

One of the most widely used techniques for modular CLP antigen display has been through chemical conjugation, which is compatible with most capsid backbones. A common approach is through cross-linking of lysine residues on the CLP surface to cysteine residues present or incorporated into the antigen [45,46,52]. This vaccine design has been shown to induce high titers of antigen-specific antibodies, and several vaccines have shown promising results in preclinical studies, as well as in clinical testing (e.g., Nicotine-Qβ [53]). However, the CLP surface often contains multiple lysine residues, resulting in uneven antigen distribution, and little control over antigen orientation. In addition, introduction of a reactive cysteine may cause antigen misfolding [54,55]. In some cases, chemical conjugation can lead to destabilization of the CLP, and cannot always facilitate high-density antigen display, due to suboptimal coupling efficiency [46,56]. Some of the limitations associated with standard chemical conjugation have been resolved by the development of click chemistry [35,48,57]. This method uses the incorporation of unnatural amino acids, allowing for increased control over site-specificity. This technique requires minimal change to the VLP and antigen, while providing a highly specific and fast coupling reaction. However, the scalability of this technology remains a concern [33,58].

#### 2.2.2. Affinity-Based Conjugation

Affinity-based conjugation systems, such as streptavidin/biotin [42,49,59], and to a lesser extent His-tag/Ni-NTA [50], have been used to facilitate unidirectional display. This is due to the single attachment site on each antigen and promotes an even display of heterologous epitopes. In addition, these methods have the advantage of being able to display larger and complex protein antigens, enabling the induction of broad, polyclonal humoral responses without compromising CLP formation and stability. For further development of the streptavidin/biotin conjugation system, engineered monomeric streptavidin (mSA) was used for antigen display on HPV CLPs containing a biotin acceptor site (AviTag^TM^) [60]. A potential disadvantage of this strategy, is that the coupling is based on a non-covalent interaction, and thus there is a risk of antigen disengagement. This can affect the coupling efficiency and diminish antigen density. Additionally, it has been hypothesized that dissociation due to an altered chemical environment in vivo, may reduce the biological efficacy of the vaccine.

#### 2.2.3. Split-Protein (Tag/Catcher) Conjugation

Split-protein (Tag/Catcher) conjugation systems have been developed [61,62,63,64,65,66,67,68,69,70] and used for covalent anchoring of vaccine antigens onto CLPs [39,51]. The split-protein technology is based on the separation of a bacterial pili protein, into a reactive peptide (Tag) and corresponding protein binding partner (Catcher). Upon mixing in solution, the Tag and Catcher rapidly react to form a spontaneous isopeptide bond [61]. In the following section, we discuss key features of the Tag/Catcher-AP205 technology and emphasize the versatility of this platform, as well as its ability to effectively mediate highly immunogenic antigen display.

The Tag/Catcher-AP205 platform was developed by genetic incorporation of the SpyTag [39] and SpyCatcher [39,51] into the capsid protein of the *Acinetobacter phage* AP205, yielding particles of 180 subunits with a diameter of approximately 36 nm and 43 nm, respectively. Since its development, the Tag/Catcher-AP205 platform has been utilized to display structurally and functionally diverse vaccine antigens, ranging in size from small peptides (e.g., toxins of 19 amino acids [71]) to large (>300 kDa) trimeric proteins [72]. These studies have repeatedly demonstrated a remarkable ability to achieve complete and even decoration of the CLP surface, with coupling efficiencies reaching 100% for smaller vaccine antigens, which are not limited by steric hindrance. Importantly, the resultant CLP-display induces antibody titers of high quality [55], affinity [41] and avidity [39]. The platform is additionally capable of effectively overcome B-cell tolerance and induce strong antibody responses against a variety of self-antigens, including IL-5, CTLA-4, PD-L1 and Her2 [39,41].

Several features of the AP205 CLP make it attractive as a vaccine backbone, including its structure, intrinsic immunogenicity and manufacturability [73,74]. While the overall capsid structure within the RNA bacteriophage family is similar, the surface exposed regions available for genetic fusion differ substantially [74,75]. The AP205 capsid is remarkable in that both the N- and C- termini are surface exposed and evenly distributed on the assembled CLP [74]. Moreover, AP205 CLPs tolerate genetic fusion at both the N- and C-terminus of the subunit protein, while maintaining stable CLP assembly [39,44]. For future large-scale manufacturing and clinical development, the Tag/Catcher-AP205 platform can be cost-effectively produced at very high yield in *E. coli* [39,74]. In fact, although the scalability of the platform has previously been questioned [76,77], our results show that fermentation can enable the production and purification of correctly assembled CLPs in the scale of grams per liter bacterial cell culture (manuscript in preparation).

#### Combinatorial Antigen Display

The ability to simultaneously display multiple different antigens on the same CLP could have numerous applications, but has so far proven technically challenging, with only a few examples reported [48,78]. However, the unique exposure of both termini of AP205 has made it possible to readily achieve such combinatorial antigen display. A vaccine targeting both HPV and placental malaria was recently described [79]. In this study, concatenated RG1 epitopes (from the HPV L2 protein) were genetically fused to the C-terminus of AP205, while VAR2CSA (a placental malaria antigen) was conjugated via the Tag/Catcher system to the N-terminus of AP205, without hampering vaccine stability. Vaccination induced high titers of functional antibodies targeting both components, thus providing a proof-of-concept for dual antigen display on the Tag/Catcher-AP205 platform.

#### Control over Antigen Orientation

From the early years of VLP research, the importance of ordered antigen display has been noted [80]. Since then, there has been a further appreciation of the benefits of unidirectional display, which can be achieved with the Tag/Catcher-AP205 technology. This was demonstrated by a study comparing different platforms presenting the malaria Pfs25 antigen with varying degrees of antigen organization [55]. The unidirectional display, obtained by the Tag/Catcher-AP205 technology, induced antibodies of higher biological efficacy, compared to when the antigen was presented in several different orientations, as the result of chemical cross-linking [55]. On that basis, it was hypothesized that unidirectional antigen display can enable induction of a more focused antibody response. Moreover, unidirectional presentation may also be exploited to mask certain regions of an antigen. In a recent study by Escolano et al., a Spy-tagged HIV envelope protein was displayed on SpyCatcher-AP205 CLPs [72]. Here, the dense unidirectional antigen display promoted induction of broadly neutralizing antibodies (bNAb), while masking dominant non-neutralizing epitopes, present near the CLP surface. An additional benefit of the Tag/Catcher conjugation technology is the small size of the SpyTag (13 amino acids), which allows its incorporation into internal antigen positions (e.g., in flexible loops) [81,82]. This provides further opportunities to optimize antigen orientation on the CLP surface.

#### Multimeric Antigen Display

Many viral antigens, such as the HIV envelope trimer, are multimeric glycoproteins [83]. Broadly neutralizing antibodies often target non-linear, conformational epitopes located at the intersection between protomers [83,84,85]. For induction of such bnAbs, the antigen thus needs to be delivered in its native quaternary structure [86]. The first study to achieve this through the Tag/Catcher technology, successfully displayed HIV envelope trimers on AP205 [72]. This demonstrates the platform’s ability to allow antigen multimerization while providing increased stabilization of the protein complex on the CLP surface. Importantly, such display enables conformational epitopes to be presented in a native-like structural context. This represents an interesting new development for the display of multimeric and highly complex antigens, for a more focused immune activation.

#### Practicality of the Tag/Catcher-AP205 System

It is possible that affinity-based platforms such as mSA/AviTag^TM^ [60] and His/trisNTA [50] could provide the same opportunities for controlled high density and unidirectional antigen display, as the split protein (Tag/Catcher) technology. However, practical aspects including versatility and ease of use favor the Tag/Catcher-AP205 platform. In our experience, SpyTag and SpyCatcher can more easily be genetically fused to a variety of antigens and allow expression in a greater range of expression systems (e.g., insect [39,41], mammalian [unpublished] and bacterial [79]), compared to mSA [60]. In addition, SpyTag is less likely to negatively affect the fold of the recombinant fusion protein. SpyCatcher has also shown to positively affect the solubility and yield of certain antigens that are otherwise difficult to express [39]. Importantly, the Tag/Catcher reaction is compatible with a broad range of buffer conditions, enabling ample opportunities to optimize antigen/CLP formulation. The recently described Spy&Go method furthermore facilitates protein purification via the SpyTag, thereby enabling a more simple antigen design without the need for a separate purification tag [67]. These practical aspects of the Tag/Catcher-AP205 technology further strengthens the future opportunities of this platform.

## 3. Prospects for Further Development of Tag/Catcher-Based CLP Display Technologies

The Tag/Catcher conjugation system is able to fulfill many of the criteria important for obtaining a viral-like epitope display, as highlighted in Table 1. This was demonstrated through the development and success of the SpyTag/SpyCatcher-AP205 platform. However, there are more opportunities to explore. Adaptations in the modular vaccine design, such as the choice of backbone, particle size and antigen density, as well as the addition of appropriate intrinsic and extrinsic adjuvants, could aid in broadening the development of CLP-based vaccines. Additionally, practical aspects such as manufacturability, scalability and cost-effectiveness, can determine future downstream success, and therefore need to be taken into account during vaccine development. In the section below, we will discuss several aspects that should be considered when designing future Tag/Catcher CLP-based vaccines and highlight several unanswered questions and hypotheses that are yet to be experimentally validated.

### 3.1. CLP Backbones

The high diversity of well-characterized viral capsids has provided a range of CLP backbones with varying structural and biological properties. The most common include CLPs derived from animal viruses (e.g., Hepatitis B core antigen (HBcAg) and HPV), plant viruses (e.g., Cowpea mosaic virus (CPMV) and tobacco mosaic virus (TMV)) and bacteriophages (e.g., MS2, Qβ and AP205) [87,88,89]. Recently, successful expression and assembly of 80 novel RNA bacteriophage CLPs was described, which further broadens the pool of potential vaccine backbones [90]. The simple structure of phage capsids makes them well suited for rapid and cost-effective recombinant bacterial expression [44,90,91]. In contrast, CLPs derived from mammalian viruses often require more complex expression systems, such as yeast [17] (Gardasil󠆿^®^) and insect cells [92] (Cervarix^®^). However, advancements in transient plant expression may offer a safe and inexpensive alternative to these conventional systems, as it allows for post-translational modifications, such as glycosylation. Thus, several complex CLPs, including HPV, have successfully been produced in plants [93,94,95,96].

Different capsid backbones may not only possess different opportunities for antigen-display but may also vary in their intrinsic immune-stimulatory qualities. This can additionally be impacted by the expression system employed [97] or engineered onto the CLP [57]. As an example, T-cell epitopes on the HPV CLP has been shown to contribute to the high immunogenicity of the particle [14,98]. Likewise, the genetic fusion of T-cell epitopes onto CLPs can induce priming of cytotoxic T-cell responses in vivo [59,99,100]. The lumen of CLPs can also be exploited for the effective delivery of intrinsic adjuvants, such as CpG [101]. Additionally, ssRNA bacteriophages such as AP205, are able to encapsulate host RNA during recombinant bacterial expression, which adjuvant the immune response via Toll-like receptor (TLR) 7 and 8 activation [74]. One recent study demonstrated that the origin of the packaged RNA can further modulate the humoral response by directing IgG class switching [97]. While prokaryotic RNA induces a predominantly IgG2 response, eukaryotic RNA elicits an IgG1 dominated response. However, packaged nucleic acids could be considered disadvantageous for further clinical development. Importantly, in the context of developing self-antigen based vaccines for the treatment of non-infectious diseases, the packaged RNA may possess a safety risk by promoting activation of an autoimmune T-cell response [102,103]. It has also been a concern, that pre-existing anti-capsid immunity could have an immunosuppressive effect on the displayed antigen [104,105]. Together, these factors indicate that it would be valuable to exploit Tag/Catcher-based antigen display on a broader range of CLP backbones.

### 3.2. Particle Size, Valiancy and Spacing

It is well documented that the pharmacokinetics of nanoparticles and their engagement with the innate immune system is affected by the particle size, charge and surface properties [106,107,108]. While large particles (500 nm–1 μm) need to be processed by antigen-presenting cells prior to transport to the lymph nodes, pathogens and VLPs of 20–200 nm have the ability to rapidly and effectively traffic through the lymphatic system in a cell-free state, and thus interact directly with B-cells in the lymph nodes [19,23,109]. However, it remains to be clarified whether variation in size within this “optimal” range, as well as the resultant valency, will affect the immune response. Thus, it is unknown if the particulate size/valency differences between e.g., HPV CLPs (55 nm particle of 360 subunits), bacteriophage CLPs such as AP205 and Qβ (30 nm particles of 180 subunits), and smaller nanoparticles such as ferritin (12 nm) [110] are significant.

The close spacing of repetitive epitopes is believed to be a key determinant for the high immunogenicity of viruses and VLPs [18,23,111,112,113]. Accordingly, antigen density has become a common quality measure for modular CLP vaccines. Early studies concluded that narrow epitope spacing of 5–10 nm is a critical determinant in humoral responses (i.e., by facilitating B-cell receptor crosslinking and B-cell activation). However, these first studies were based on hapten-polymer conjugates as repetitive antigens [111,114]. It was additionally shown that the immunogenicity of the native VSV-G protein displayed at high density by the enveloped RNA virus (VSV) was significantly higher compared to less organized forms of recombinant VSV-G protein (i.e., soluble or displayed in micelles) [80]. A study using HBc and Qβ CLPs has since demonstrated a positive correlation between antigen density and the vaccine-induced antigen-specific IgG responses, and observed that high doses of low-density particles could not counteract this effect [115]. However, antigen conjugation in this study was achieved using chemical cross-linking and thus the maximum coupling density tested did not exceed 50%. Given the ability of the Tag/Catcher-based conjugation system to achieve complete decoration of CLP backbones, we propose to use this technology to further investigate whether there exists a threshold, beyond which increasing the density would have no further effect. This could have specific implications in the optimization of modular vaccines, e.g., when greater antigen spacing is required for providing sufficient access to epitopes near the capsid surface [116].

### 3.3. Effect of Platform Rigidity

The influence of platform rigidity is also a topic of debate, and it is yet to be confirmed whether epitopes that are held as part of a rigid, semi-crystalline structure (e.g., HPV) possess the same immunological properties as epitopes that are part of a more flexible structure (e.g., modular CLPs). If this is the case, the use of long flexible linkers involved in some of the more complex conjugation strategies should be avoided. Likewise, the nature of the underlying VLP should be considered. There are several examples where enveloped VLPs and other lipid-based vaccine platforms have successfully been used for induction of protective immune responses; targeting either the native surface protein (e.g., the hepatitis B virus (HBV) surface antigen (HBsAg) [117]) or a displayed foreign antigen [118,119,120]. Recently, two studies have performed head-to-head comparisons between lipid- and capsid-based VLP vaccines. Chen et al. showed that vaccination with a self-antigen peptide displayed on Qβ CLPs resulted in a 200-fold increase in antigen-specific IgG titers, compared to antigen presentation on liposome particles [113]. In contrast, Marini et al. compared Pfs25 antigen display via the Tag/Catcher conjugation system on either HBsAg lipid-based particles or AP205 CLPs, and showed no significant differences in antibody titers and functionality between the two vaccines [77]. The duration of the induced antibody responses was however not tested in either study. This could be an important factor to investigate, as the antibody longevity of licensed VLP-based vaccines differ substantially. When targeting many important pathogens, such as HIV, HPV and malaria, neutralizing antibody levels need to be maintained in order for the vaccine to be protective. This is due to the importance of preventing the initial infection of these pathogens. In such cases, memory B-cell dependent humoral responses would not be sufficient [121]. While the HBV vaccine has shown to be effective and has decreased HBV prevalence worldwide [122], the long-term efficacy of the HBV vaccine is memory B-cell dependent. In general, durable antibody responses are not induced, even after three doses [123]. The RTS,S vaccine, which is based on genetic incorporation of a malaria antigen into HBsAg VLPs, only showed short-term efficacy in clinical trials, due to a similar rapid decline in antibody levels [118,124,125]. In contrast, HPV CLPs are strong inducers of long-lived antibody-producing plasma cells [14,126]. These marked differences in the induced immune responses could reflect fundamental differences in the immunological properties of lipid- versus capsid-based backbones [127].

### 3.4. The Need for Thorough Comparative Studies

As described above, the immune response elicited by CLP-based vaccines is likely influenced by multiple factors. However, direct comparisons of many of these variables have so far been lacking in the field. Firstly, there are no conclusive comparisons between the different conjugation systems. A study by Leneghan et al. indicates that unidirectional antigen display obtained by split-protein conjugation is more favorable than the disordered display elicited by chemical conjugation [55]. However, these results may in part be due to a combination of additional confounding factors such as the particle size, epitope density and chemical modifications of the antigen, all of which varied between the tested vaccines. This study highlights the difficulty with conducting such comparative studies, as it is challenging to separate the many variables intrinsically associated with different CLP platforms. Likewise, the full immunological effect of capsid backbones from divergent origins, containing different intrinsic properties, remains to be defined. When considering the final vaccine formulation, it would moreover be important to compare the effect of extrinsic adjuvants, to ascertain which work best in synergy with the immune-activation obtained by the CLP platform. Improved consensus in these areas would greatly aid in refining and directing the rational design of CLP-based vaccines. This could provide a toolbox of components that can intentionally be combined to modulate or skew the response, to induce the most suitable immune activation against a given disease or antigen.

## 4. Concluding Remarks

Overall, CLP-based display has proven effective in increasing the immunogenicity of vaccine antigens. This has offered new possibilities for developing vaccines against important infectious diseases, which have proven difficult to target using conventional vaccine strategies. CLP-display has even enabled the induction of strong antibody responses to self-antigens, and thus made it possible to develop therapeutic vaccines against non-infectious diseases, including cancer.

In this review, we have highlighted the unique ability of the split-protein-based Tag/Catcher-AP205 platform to mediate high-density, unidirectional antigen presentation of a broad range of vaccine antigens. We believe this highly versatile technology, combined with the recent discovery of many novel CLP backbones, will create numerous opportunities for strategic design of new CLP vaccines with distinct immune-stimulatory properties.

## Figures and Tables

**Table 1:** Properties of different conjugation strategies used for capsid-like particles (CLP) antigen display.

	Antigen Display				Versatility			Examples
	High Density	Covalent linkage	Uniform Distribution	Unidirectional	Antigen Size/Complexity	Expression Systems	Capsid Backbones	
Genetic Fusion	+	+	+	+	−	−	+	[31,44]
Chemical Conjugation	−	+	−	+/−	+/−	+	+	[45,46]
Click Chemistry	+/−	+	+	+	+/−	+	+	[47,48]
Affinity-based	+/−	−	+	+	+	+/−	+	[39,49,50]
Split-protein Systems	+	+	+	+	+	+	+/−	[39,41,51]

+ indicates property can be achieved, − indicates property not readily achieved, +/− indicates property can only partly be achieved or has not been experimentally validated.
